# Systemic complement factors in aging, Alzheimer’s disease and other dementias: a longitudinal study over 10 years

**DOI:** 10.1186/s13024-026-00927-3

**Published:** 2026-01-12

**Authors:** Xiaofeng Fu, Huimin Cai, Shuiyue Quan, Weiyun Zhang, Yumei Geng, Qing Tian, Ziye Ren, Yinghao Xu, Chengyu An, Jiaqi Li, Changbiao Chu, Wei Wang, Yana Pang, QianQian Wang, Lu Lu, Qi Wang, Yan Li, Fangyu Li, Shuya Nie, Longfei Jia

**Affiliations:** Department of Neurology & Innovation Center for Neurological Disorders, Xuanwu Hospital, National Center for Neurological Disorders, Capital Medical University, Beijing, 100053 China

## Abstract

**Background:**

Complement dysregulation is increasingly recognized in Alzheimer’s disease (AD). However, the temporal profile of complement alterations preceding AD onset and their distinction from age-related immune changes remain poorly defined. Clarifying these dynamics could provide insights into AD pathogenesis and identify systemic factors that predict disease onset and progression.

**Methods:**

We conducted a study involving two cohorts: a longitudinal cohort (*n* = 235; all cognitively normal at baseline) and a cross-sectional cohort (*n* = 323; including 53 with AD, 54 with vascular dementia, 51 with Parkinson’s disease dementia, 56 with behavioral variant frontotemporal dementia, and 52 with dementia with Lewy bodies). Plasma levels of 14 complement factors were assessed every 2 years over a 10-year follow-up period in the longitudinal cohort and once in the cross-sectional cohort.

**Results:**

In the longitudinal cohort, aging was accompanied by gradual reductions in C4, C4b, Factor I, and Properdin and by increases in Factor D. These changes were more pronounced in individuals who subsequently developed AD. Importantly, this pattern of complement alterations was detectable during the preclinical and clinical phases of AD but was not observed in other dementias. In the cross-sectional cohort, the same complement profile was specific to AD and distinguished it from other dementia subtypes.

**Conclusions:**

The results of this study indicate an AD-specific peripheral complement signature associated with disease development, highlighting complement factors as critical immune mediators that link aging and AD. This signature implicates complement factors as promising systemic markers for early detection and potential therapeutic targeting in preclinical AD.

**Supplementary Information:**

The online version contains supplementary material available at 10.1186/s13024-026-00927-3.

## Introduction

Alzheimer’s disease (AD) is the most common age-related neurodegenerative disease and the leading cause of dementia among older adults worldwide [1]. As global life expectancy rises, the burden of dementia continues to grow, with approximately 55 million affected individuals and a projected increase to 78 million by 2030 and 139 million by 2050 [2]. Amyloid-β (Aβ) and phosphorylated tau are the core pathological hallmarks of AD, and accumulating evidence indicates that immune-related inflammatory processes also play an important role in AD pathogenesis [3, 4].

The complement system, an essential component of innate immunity, contributes to pathogen clearance, removal of apoptotic cells, and elimination of misfolded proteins [5]. Within the central nervous system (CNS), complement factors are actively involved in neuronal development, synaptic remodeling, and immune surveillance [6]. However, aberrant complement activation is increasingly associated with neuroinflammatory pathologies, including AD. Immunohistochemical studies of the brains of patients with AD have shown that classical complement pathway proteins, including C1q, C3, and C4, co-localize with amyloid plaques and neurofibrillary tangles [7–9]. Genetic studies further reinforce this link by identifying AD-associated variants in complement-related genes, such as complement receptor 1 (CR1), C1S, and Factor H [10]. These findings indicate that complement factors contribute to AD progression and may represent targets for early diagnosis and intervention. However, the temporal dynamics of complement activation during aging and its specific association with AD pathophysiology remain to be fully understood. Previous work has predominantly relied on cross-sectional designs or single time-point measurements, limiting the ability to disentangle age-related immune alterations from disease-specific trajectories. Given the pronounced inter-individual variability of complement factors and the prolonged preclinical course of AD, longitudinal mapping of complement factors is essential for distinguishing pathological aging from benign immune senescence.

AD predominantly affects older individuals, with age being the strongest established risk factor. Aging and AD share multiple molecular and cellular features, particularly immune dysregulation and chronic low-grade systemic inflammation, including disturbed complement activation. Population-based and experimental studies have shown age-related declines in complement fators such as C3 and C4, potentially fostering a pro-inflammatory immune microenvironment, compromising pathogen clearance, and contributing to cumulative tissue injury [11–13]. These observations suggest that complement dysregulation constitute an important immunological link between aging and AD pathogenesis. However, it remains unclear whether age-related alterations in the complement system directly predispose individuals to AD. Most evidence linking systemic complement alterations to AD is from cross-sectional studies, which provide limited insight into the temporal dynamics of complement activity during disease development. Given the slow and clinically silent progression of AD, which may begin a decade or more before symptom onset [14], longitudinal assessments are essential to evaluate how complement dysregulation evolves during the preclinical stages of disease. Complement changes have also been reported in other dementias, including vascular dementia (VaD), Parkinson’s disease dementia (PDD), behavioral variant frontotemporal dementia (bvFTD) and dementia with Lewy bodies (DLB) [15–18]. Determining whether complement activation patterns are specific to AD or represent a shared feature of neurodegeneration is critical for identifying disease-specific mechanisms and therapeutic targets.

To address these gaps, we conducted a 10-year longitudinal study examining complement factors across classical (C1q, C2, C4, and C4b), alternative (Factor B, Factor D, Factor H, Factor I, and Properdin), lectin (mannose-binding lectin [MBL]), and terminal (C5, C5a, C9, and C5b-9) pathways. Complement trajectories were assessed in cognitively normal controls and compared with those of individuals with preclinical AD (Pre-AD). Cross-sectional comparisons of patients diagnosed with VaD, PDD, bvFTD, and DLB were also conducted. In this study, we aimed to delineate the complement dynamics specifically associated with AD pathogenesis and identify potential complement factors that predict disease onset and progression.

## Results

### Participant characteristics

**Fig. 1:**
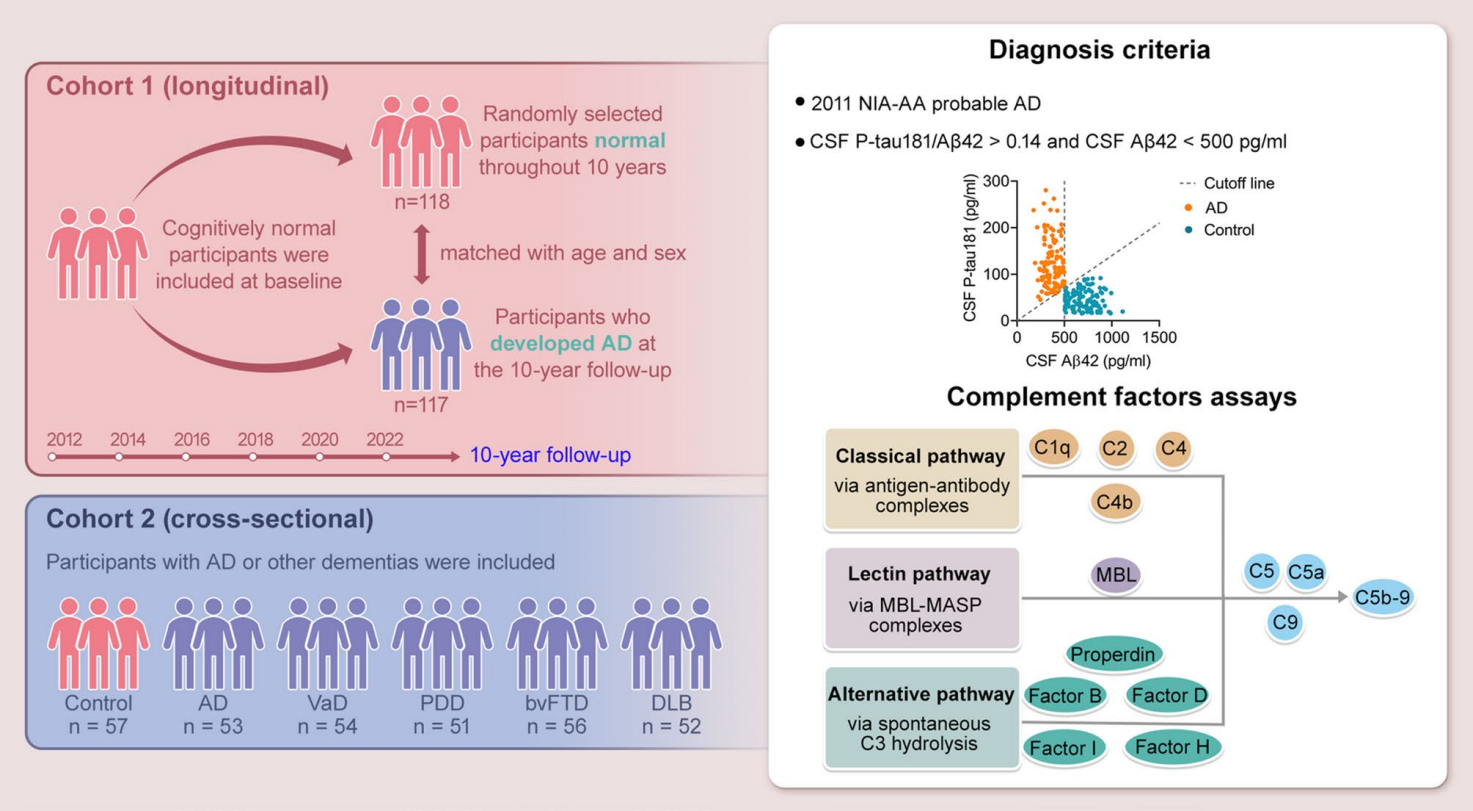
Schematic representation of the study design. A longitudinal cohort initiated in 2012 and an independent cross-sectional cohort were included in this study. In the longitudinal cohort, cognitively normal participants were followed for 10 years, with plasma complement factor levels assessed every 2 years to investigate temporal dynamics associated with AD progression. The cross-sectional cohort comprised individuals with AD or other types of dementia. The scatter plot illustrates CSF biomarker thresholds, specifically the P-tau181/Aβ42 ratio (0.14) and Aβ42 levels (500 pg/mL), used to differentiate patients with AD from cognitively normal controls. The orange dots represent participants with AD, and blue dots indicate cognitively normal participants. Complement factors from the classical, lectin, and alternative pathways were profiled to evaluate systemic immune changes. Aβ, amyloid-β; AD, Alzheimer’s disease; bvFTD, behavioral variant frontotemporal dementia; CSF, cerebrospinal fluid; C5b-9, complement membrane attack complex; DLB, dementia with Lewy bodies; MBL, mannose-binding lectin; MASP, MBL-associated serine protease; NIA-AA, the National Institute on Aging-Alzheimer’s Association; VaD, vascular dementia; PDD, Parkinson’s disease dementia; P-tau, phosphorylated tau

We used two independent cohorts in this study (Fig. 1). Detailed demographic and clinical data for cohort 1 and cohort 2 are presented in Tables 1 and 2. Patients with AD did not differ significantly from controls in age, sex, or years of education. By contrast, the AD groups in both cohorts had a higher prevalence of *APOE ε4* carriers and lower Mini-Mental State Examination (MMSE) scores than the control groups (all *P* < 0.05). In cohort 2, MMSE scores were also significantly lower in patients with VaD, PDD, bvFTD and DLB than in controls (all *P* < 0.05). Furthermore, compared with control participants, individuals with AD showed significantly reduced cerebrospinal fluid (CSF) Aβ42 levels and increased T-tau and P-tau181 levels (all *P* < 0.05). In cohort 1, total plasma C3 concentrations were lower in Pre-AD at baseline and further reduced in AD at the 10-year follow-up compared with cognitively normal controls (Supplementary Table S1, all *P* < 0.05). To address the possibility of subclinical inflammation more directly, we measured high-sensitivity C-reactive protein (hsCRP) in cohort 1; hsCRP concentrations were low and showed no significant differences between cognitively normal controls and individuals with Pre-AD at baseline, or between controls and AD at the 10-year follow-up, indicating comparable systemic inflammatory burden across groups (Supplementary Table S1, all *P* > 0.05).

**Table 1:** Characteristics of participants in cohort 1

		Baseline		At the 10-year follow up	
Characteristic	Total Sample (Follow up, *n* = 235)	Control (*n* = 118)	Pre-AD (*n* = 117)	Control (*n* = 118)	AD (*n* = 117)
Age, mean (SD), year	71.8 (7.4)	61.3 (7.4)	62.3 (7.4)	71.3 (7.4)	72.3 (7.4)
Educational attainment, mean (SD), year	9.2 (2.0)	9.2 (2.0)	9.2 (2.0)	9.2 (2.0)	9.2 (2.0)
Women, No. (%)	118 (50.2)	59 (50.0)	59 (50.4)	59 (50.0)	59 (50.4)
*APOE ε4* status, No. (% positive)	69 (29.4)	21 (17.8)	48 (41.0)^*^	21 (17.8)	48 (41.0)^*^
MMSE score, mean (SD)	24.7 (4.5)	30.0 (0)	29.8 (0.4)	29.0 (0.6)	20.4 (2.0)^*^
CSF biomarkers, mean (SD)					
Aβ42, pg/ml	537.6 (202.6)	/	/	705.8 (136.6)	367.9 (78.6)^*^
T-tau, pg/ml	464.4 (190.8)	/	/	342.2 (102.5)	587.7 (179.8)^*^
P-tau181, pg/ml	83.3 (56.4)	/	/	45.1 (21.0)	124.0 (51.9)^*^

The values of age, education year, MMSE, Aβ42, T-tau, and P-tau are shown as mean (SD). AD, Alzheimer’s disease; Pre-AD, preclinical Alzheimer’s disease; ApoE ε4, apolipoprotein ε4; MMSE, Mini-Mental State Examination; SD, standard deviation. *P < 0.05 compared to controls

**Table 2:** Characteristics of participants in cohort 2

Characteristic	Total Sample (*n* = 323)	Control (*n* = 57)	AD (*n* = 53)	VaD (*n* = 54)	PDD (*n* = 51)	bvFTD (*n* = 56)	DLB (*n* = 52)
Age, mean (SD), year	68.9 (6.4)	70.6 (7.0)	69.5 (6.8)	70.0 (6.1)	68.5 (6.4)	68.3 (5.0)	67.7 (7.1)
Educational attainment, mean (SD), year	8.9 (2.2)	8.8 (2.6)	8.6 (2.3)	8.7 (2.2)	8.7 (2.0)	9.2 (2.1)	9.1 (2.0)
Women, No. (%)	164 (50.8)	29 (50.9)	27 (50.9)	26 (48.1)	26 (51.0)	29 (51.8)	27 (51.9)
APOE ε4 status, No. (% positive)	77 (23.8)	10 (17.5)	22 (41.5)^*^	14 (25.9)^*^	10 (19.6)	11 (19.6)	10 (19.2)
MMSE score, mean (SD)	22.3 (2.4)	29.0 (0.6)	20.5 (3.1)^*^	20.8 (3.4)^*^	21.2 (3.1)^*^	20.6 (3.0)^*^	21.1 (2.5)^*^
CSF biomarkers, mean (SD)							
Aβ42, pg/ml	604.4 (176.1)	711.1 (147.1)	365.3 (67.6)^*^	650.0 (108.0)	655.6 (167.3)	605.9 (172.9)	631.6 (133.9)
T-tau, pg/ml	426.2 (144.5)	316.4 (87.6)	599.2 (179.4) ^*^	367.9 (78.5)	379.0 (74.2)	459.9 (106.0)	440.7 (121.6)
P-tau181, pg/ml	62.5(34.5)	49.6 (26.2)	120.3 (44.0)^*^	50.7 (13.4)	49.2 (10.8)	48.8 (10.5)	57.2 (13.6)

The values of age, education year, MMSE, Aβ42, T-tau, and P-tau are shown as mean (SD). AD, Alzheimer’s disease; VaD, vascular dementia; PDD, Parkinson’s disease dementia; bvFTD, behavioral variant frontotemporal dementia; DLB, dementia with Lewy body; ApoE ε4, apolipoprotein ε4; MMSE, Mini-Mental State Examination; SD, standard deviation. *P < 0.05 compared to controls

### Plasma levels of complement factors in AD

**Fig. 2:**
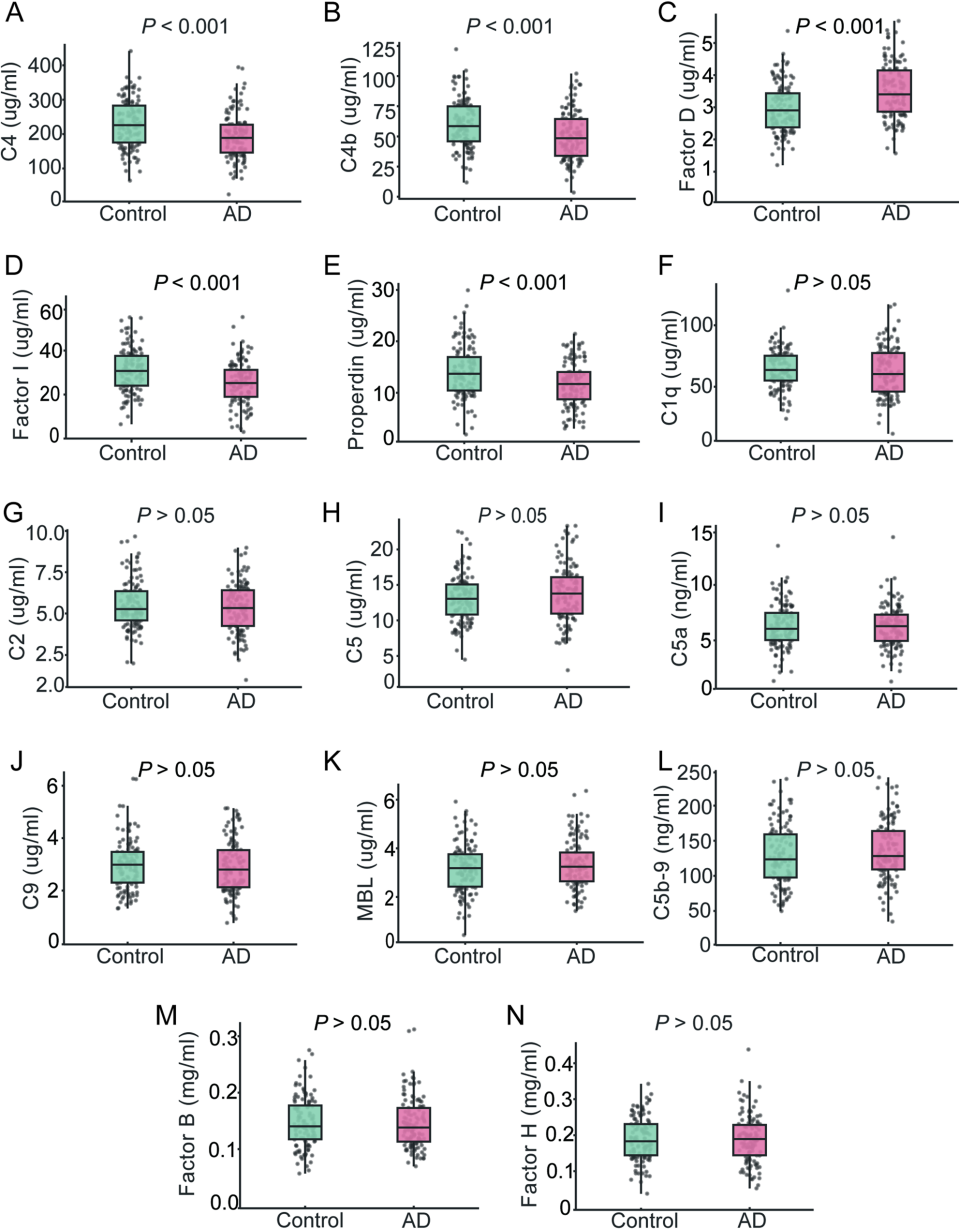
Levels of plasma complement factors at the 10-year follow-up. Plasma levels of complement C4 (**A**), C4b (**B**), Factor D (**C**), Factor I (**D**), Properdin (**E**), C1q (**F**), C2 (**G**), C5 (**H**), C5a (**I**), C9 (**J**), MBL (**K**), C5b-9 (**L**), Factor B (**M**), and Factor H (**N**) were measured at the 10-year follow-up. The *P* values from the *t*-test comparing participants with AD and cognitively normal controls are presented within each corresponding panel. *n* = 118 (controls), 117 (AD). AD, Alzheimer’s disease; C5b-9, complement membrane attack complex; MBL, mannose-binding lectin

In cohort 1, we measured a panel of 14 complement factors, including C1q, C2, C4, C4b, C5, C5a, MBL, C9, C5b-9, Factor B, Factor D, Factor I, Factor H and Properdin. At the 10-year follow-up, patients with AD had significantly lower plasma levels of C4, C4b, Factor I and Properdin, and higher levels of Factor D, compared with controls (all *P* < 0.001; Fig. 2A-E). The remaining complement factors (C1q, C2, C5, C5a, C5b-9, C9, MBL, Factor B and Factor H) did not differ significantly between the groups (all *P* > 0.05; Fig. 2F-N).

To further assess the discriminative capacity of these complement factors, predicted probabilities from logistic regression models were used to construct receiver operating characteristic (ROC) curves. A five-factor panel comprising C4, C4b, Factor I, Properdin and Factor D discriminated AD from controls with good accuracy (AUC = 0.805; Fig. S1A), which further improved when *APOE* status was included (AUC = 0.819; Fig. S1B). By contrast, ROC curves for individual complement components showed only modest discriminative performance (AUC = 0.631–0.671; Fig. S1C-G).

### Plasma complement factor levels in preclinical AD

**Fig. 3:**
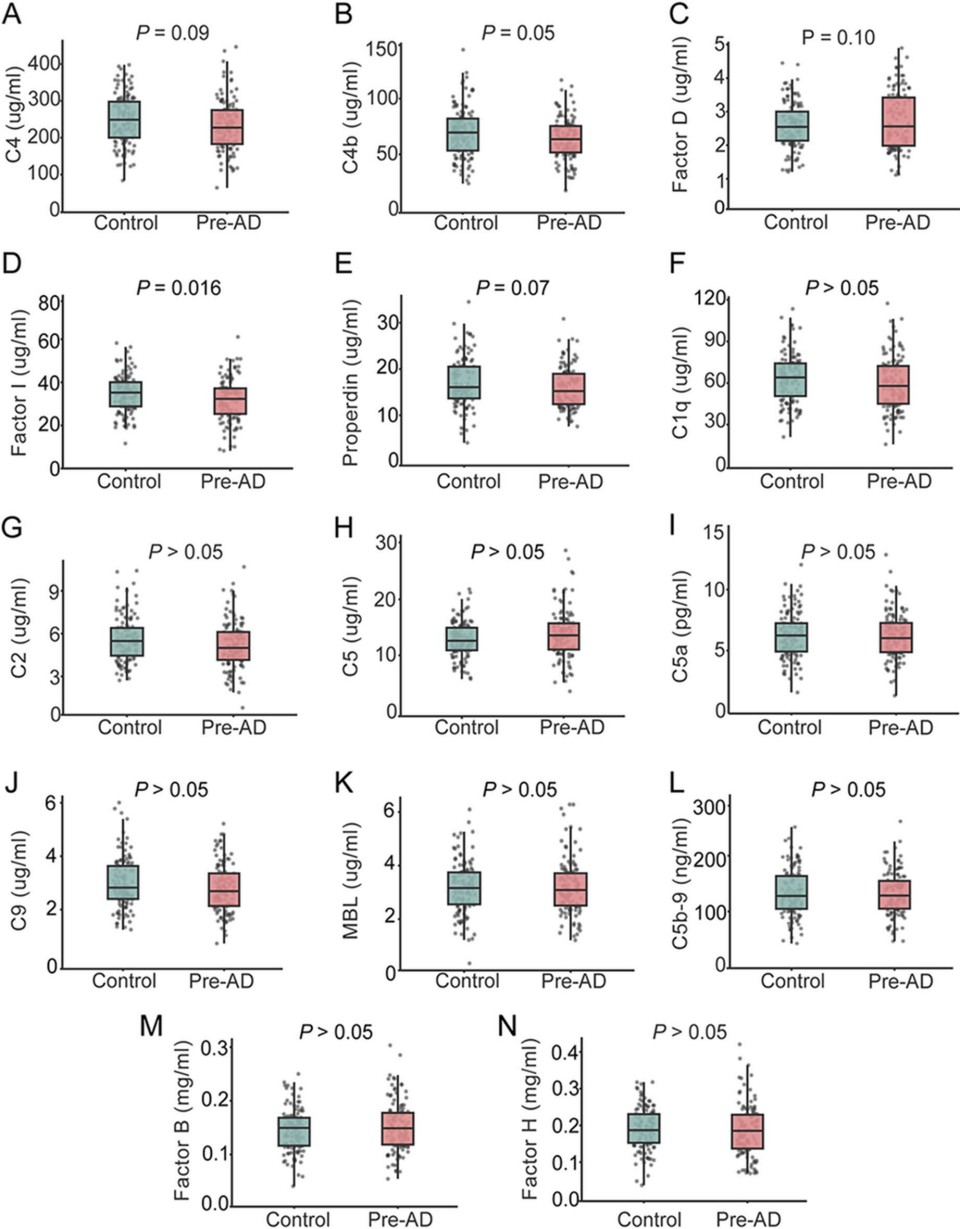
Levels of plasma complement factors at baseline. Plasma levels of complement C4 (**A**), C4b (**B**), Factor D (**C**), Factor I (**D**), Properdin (**E**), C1q (**F**), C2 (**G**), C5 (**H**), C5a (**I**), C9 (**J**), MBL (**K**), C5b-9 (**L**), Factor B (**M**), and Factor H (**N**) were measured at baseline. The *P* values from the *t*-test comparing participants with Pre-AD and cognitively normal controls are presented within each corresponding panel. *n* = 118 (controls), 117 (Pre-AD). C5b-9, complement membrane attack complex; MBL, mannose-binding lectin; Pre-AD, preclinical Alzheimer’s disease

At baseline, participants in cohort 1 with Pre-AD showed a downward trend in plasma C4 (*P* = 0.09; Fig. 3A), C4b (*P* = 0.05; Fig. 3B) and Properdin (*P* = 0.07; Fig. 3E), and an upward trend in Factor D (*P* = 0.10; Fig. 3C) relative to cognitively normal controls; none of these differences reached conventional statistical significance. By contrast, plasma Factor I was significantly reduced in Pre-AD (*P* = 0.01; Fig. 3D), identifying Factor I as the earliest and most pronounced complement alteration in this stage. No significant group differences were observed for the remaining complement factors, including C1q, C2, C5, C5a, C9, MBL, C5b-9, Factor B and Factor H (all *P* > 0.05; Fig. 3F-N).

ROC analysis showed that a five-factor panel comprising C4, C4b, Factor I, Properdin and Factor D achieved acceptable discrimination between Pre-AD and controls (AUC = 0.656; Supplementary Fig. 2A), which improved when APOE status was incorporated (AUC = 0.706; Supplementary Fig. 2B). These findings suggest that the combined model may facilitate the early identification of individuals at risk for Pre-AD up to 10 years before the onset of cognitive impairment. By contrast, individual complement factors exhibited only limited discriminative power (AUC = 0.543–0.584; Supplementary Fig. 2C-G). Given that *APOE ε4* is a major genetic risk factor for AD, we examined whether baseline complement levels differed according to *APOE ε4* carrier status. Baseline concentrations of the 14 complement factors did not differ significantly between APOE ε4 carriers and non-carriers (all *P* > 0.05; Supplementary Fig. 3). Similarly, no sex-related differences in baseline complement profiles were observed (all *P* > 0.05; Supplementary Fig. 4). Thus, in this cohort, the complement alterations described above appear largely independent of APOE ε4 status and sex.

### Longitudinal change of complement factors

We analyzed plasma samples collected at baseline and at the 2-, 4-, 6-, 8-, and 10-year follow-up visits to characterize the longitudinal trajectories of complement factors across aging and the AD course (Supplementary Figs. 5–18). For the longitudinal analyses, age, sex, years of education, *APOE ε4* status, diagnosis, visit number (follow-up time), and storage duration were all initially entered as fixed effects in the linear mixed-effects models. In the final models, only diagnosis and visit number remained significantly associated with complement levels (*P* < 0.05), whereas age, sex, education, *APOE ε4* status, and storage duration were not statistically significant (all *P* > 0.05) and were therefore excluded from the mixed-effects equation. In cohort 1, C4, C4b, Factor I and Properdin decreased significantly, whereas Factor D increased (all *P* < 0.05; Table 3). These findings indicate that complement dysregulation is not merely a signature of the aging process, but is also intimately linked to the trajectory of AD.

**Table 3:** Associations of longitudinal plasma complement factors levels with aging and AD in cohort 1

Complements	AD		Control	
	βestimate	*P* value	βestimate	*P* value
C4	-7.3866	6.75e-08	-2.6525	0.0458
C4b	-2.88820	1.04e-12	-1.71566	1.23e-05
Factor D	0.134605	< 2e-16	0.074749	1.95e-06
Factor I	-1.15503	9.28e-09	-0.40904	0.0358
Properdin	-0.92357	< 2e-16	-0.63652	2.47e-11

The β-estimates and *P* values are derived from linear mixed effects models with the interaction between time from baseline and complement factors slopes. AD, Alzheimer’s disease; C4, Complement 4; C4b, Complement 4b

**Fig. 4:**
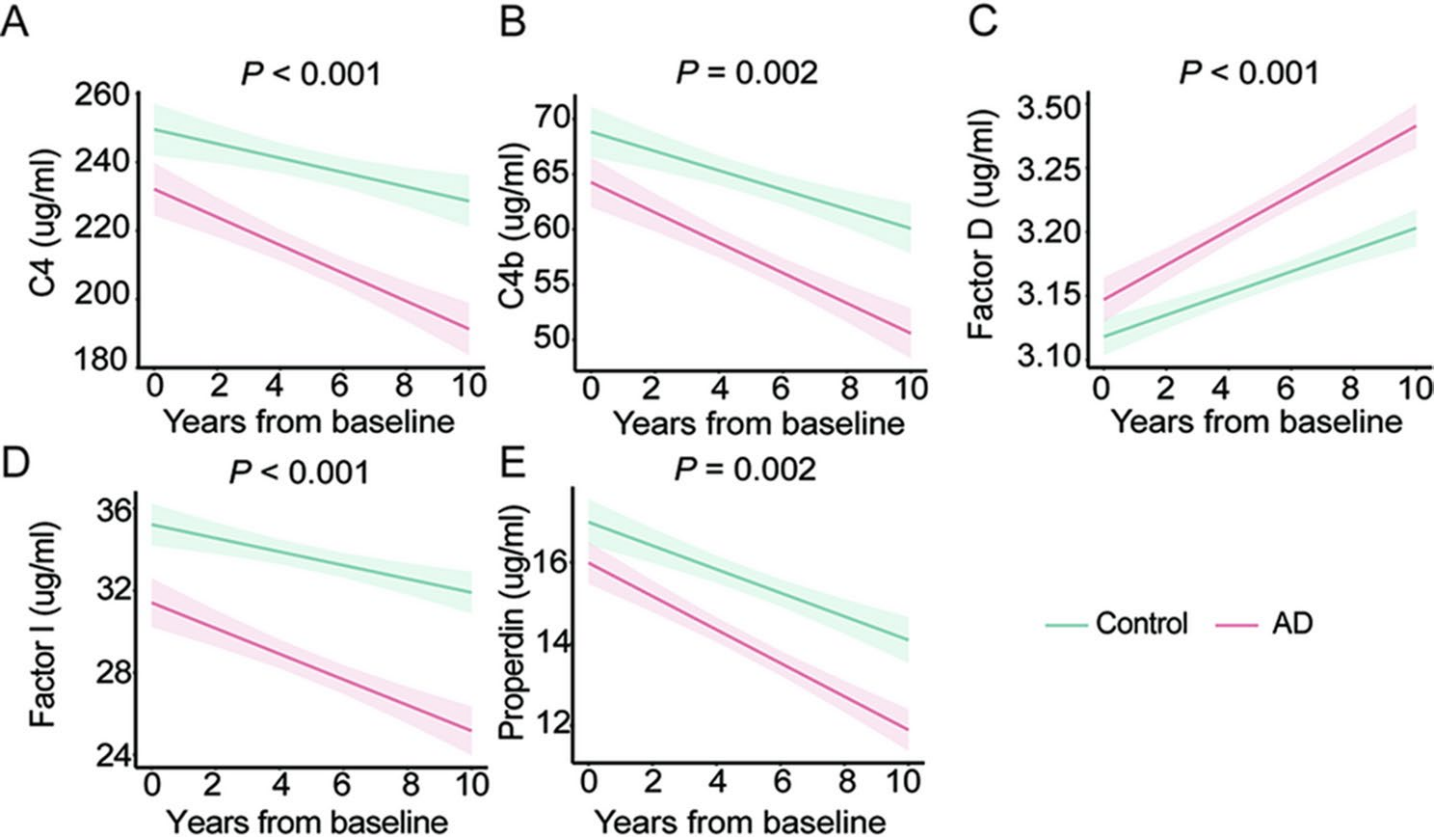
Longitudinal changes in plasma complement factors. Longitudinal trajectories of plasma complement C4 (**A**), C4b (**B**), Factor D (**C**), Factor I (**D**), and Properdin (**E**) are shown, stratified by diagnostic group. The x-axis represents time from the baseline. The shaded areas indicate the 95% confidence intervals of the fitted regression lines derived from the linear mixed-effects models, which account for the interaction between follow-up duration and diagnostic status. *n* = 118 (control), 117 (AD). AD, Alzheimer’s disease

To determine whether these trajectories differed by diagnostic outcome, we included an interaction term between follow-up time and diagnosis. The declines in C4, C4b, Factor I, and Properdin, and the increase in Factor D, were all significantly more pronounced in participants with AD than in controls (time × diagnosis interaction, *P* < 0.05; Fig. 4), consistent with an AD-specific pattern of complement remodeling superimposed on age-related changes. No significant longitudinal changes were observed for the other complement components, including C1q, C2, C5, C5a, C5b-9, C9, MBL, Factor B and Factor H (all *P* > 0.05; Supplementary Fig. 19).

### Correlation of complement factors with core AD biomarkers

**Fig. 5:**
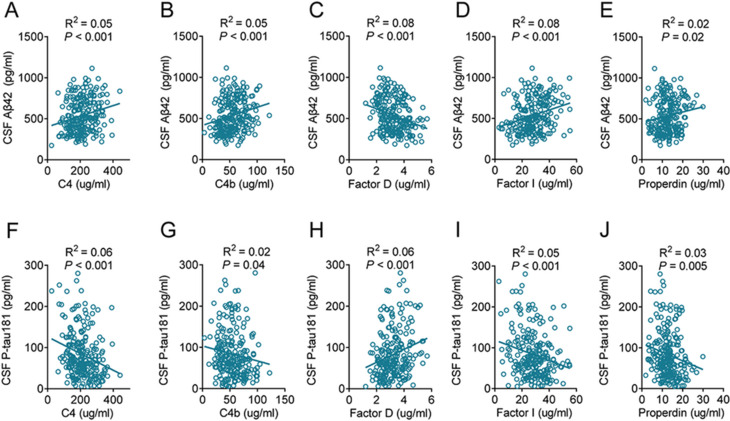
Correlations between complement factors and CSF Aβ42 and P-tau181. The levels of plasma complement C4 (**A and F**), C4b (**B and G**), Factor D (**C and H**), Factor I (**D and I**), and Properdin (**E and J**) at the 10-year follow-up were significantly correlated with CSF Aβ42 (**A-E**) and P-tau181(**F-J**). The *P* values are presented within each corresponding panel. *n* = 118 (controls), 117 (AD). Aβ, amyloid-β; AD, Alzheimer’s disease; CSF, cerebrospinal fluid

To evaluate the relationship between plasma complement factors and AD-related pathology, we performed correlation analyses between the following complement factors: C4, C4b, Factor D, Factor I, and Properdin, and core CSF biomarkers of AD. At the 10-year follow-up, all five complement factors were significantly associated with CSF Aβ42 and P-tau181 levels (all *P* < 0.05, Fig. 5), suggesting that these factors may play a role in neuropathological processes underlying AD.

### The specificity of complement factors for AD

**Fig. 6:**
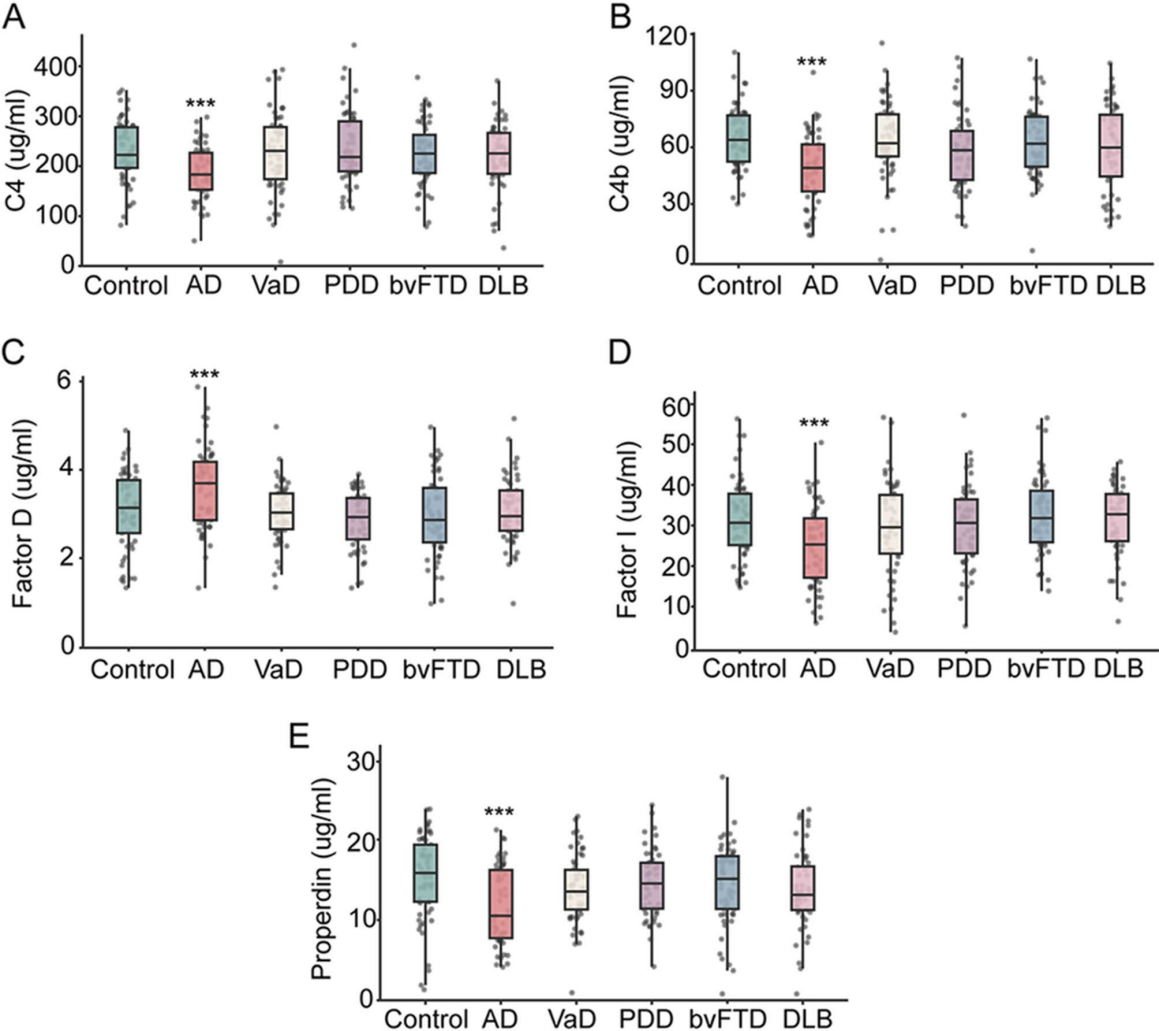
Changes in complement factors in other types of dementia. The levels of complement C4 (**A**), C4b (**B**), Factor D (**C**), Factor I (**D**), and Properdin (**E**) in cohort 2. The levels of complement factors were compared between participants with different types of dementia and cognitively normal controls using the t-test. *** *P* < 0.001. *n* = 57 (controls), 53 (AD), 54 (VaD), 51 (PDD), 56 (bvFTD), 52(DLB). AD, Alzheimer’s disease; bvFTD, behavioral variant frontotemporal dementia; DLB, dementia with Lewy bodies; PDD, Parkinson’s disease dementia; VaD, vascular dementia

To evaluate the disease specificity of these alterations, we analyzed plasma complement levels across dementia subtypes in cohort 2. Significant changes in C4, C4b, Factor I, Factor D and Properdin were observed only in participants with AD (all *P* < 0.001; Fig. 6). In contrast, the levels of these factors did not significantly differ between controls and patients with VaD, PDD, bvFTD or DLB (all *P* > 0.05; Fig. 6). Moreover, no significant differences in C1q, C2, C5, C5a, C9, MBL, C5b-9, Factor B or Factor H were detected across other dementia subtypes (all *P* > 0.05; Supplementary Fig. 20). These findings indicate that the combined pattern of C4, C4b, Factor I, Factor D and Properdin alterations represents a plasma complement signature that is specific for AD and distinguishes it from other common forms of dementias.

## Discussion

In this 10-year follow-up study, we delineated the longitudinal trajectories of key complement factors across aging and AD. C4, C4b, Factor I, Factor D and Properdin showed progressive deviations from normative aging trajectories exclusively in individuals who later converted to AD. These alterations correlated robustly with established CSF biomarkers, indicating that peripheral complement remodeling reflects AD-specific pathophysiology rather than age-related change. Collectively, these findings establish complement dysregulation as a systemic hallmark of Pre-AD and identify a discrete panel of proteins with potential for early detection and treatment.

The complement system is a core component of innate immunity and can be activated through the classical, lectin and alternative pathways. Within the classical and lectin pathways, C4 and its activation fragment C4b act as key early effectors. Our longitudinal analyses revealed a sustained decline in plasma C4 and C4b in individuals who developed AD, with reductions detectable nearly a decade before clinical onset. This trajectory is consistent with evidence of persistent C3 and CRP consumption in AD [3, 15], proteomic studies reporting reduced plasma C4 [16] and CSF studies linking lower C4 levels to higher amyloid burden [17, 18]. Histopathological analyses further support this interpretation, showing that Aβ plaques harbor C3 and C4 activation fragments that scale with lesion density and clinical severity [19]. Although elevated plasma C4 has been observed in AD [20], these opposite trends probably indicate stage-specific changes. Early decreases may result from ongoing consumption during preclinical phases, while later increases could be due to compensatory upregulation or altered production and clearance. Genetic evidence further supports a critical role for C4 in synaptic homeostasis: *C4A* copy number determines the extent of activity-dependent synaptic pruning, with reduced expression impairing pruning [21, 22], and overexpression driving excessive synapse loss in schizophrenia models [23]. Together, these findings support a dose-sensitive role for complement in synaptic pruning, whereby both insufficient and excessive C4 activity can disrupt synaptic homeostasis.

Complement regulation is equally essential for maintaining immune balance. Factor I, a serine protease that inactivates C3b and C4b in the presence of cofactors such as Factor H, C4-binding protein and CR1 [24, 25], acts as a negative regulator across the classical, lectin and alternative pathways. In our cohort, Factor I declined progressively in participants who developed AD, suggesting reduced control of complement amplification. Neuropathological studies have localized Factor I within Aβ deposits and cortical pyramidal neurons, consistent with a role in constraining local cascade activation [26]. Dysregulation of Factor I has also been reported in tauopathies [27], highlighting its relevance beyond Aβ pathology. Associations between plasma Factor I and hippocampal volume [28] further implicate this regulator in structural degeneration. Clinically, Factor I deficiency is characterized by elevated interleukin (IL)-1β and IL-6, and blockade of IL-6R has shown therapeutic benefit [29–32]. Collectively, these findings identify Factor I as a central checkpoint whose decline may couple complement overactivation with neuroinflammation and progressive brain injury.

The alternative pathway is strongly influenced by Properdin and Factor D. Properdin is the only known positive regulator, stabilizing the C3 convertase and amplifying downstream activation [33, 34]. Beyond this canonical role, Properdin can act as a pattern recognition molecule and promote proinflammatory signaling via microglial Mincle binding [35]. Cross-sectional studies have reported that elevated Properdin predicts conversion from mild cognitive impairment (MCI) to AD [36]. In our longitudinal cohort, circulating Properdin declined progressively, particularly in individuals who converted to AD, consistent with sustained consumption under chronic complement activation. Similar depletion of Properdin has been described in severe infections and sepsis, where it is associated with impaired host defence [37]. Experimental data from AD mouse models showing Properdin deposition on Aβ plaques [38] further support its involvement in AD-related neuroinflammation. Together, these observations place Properdin at the interface of systemic immunity and neurodegeneration and suggest that its trajectory in AD reflects a balance between pathological amplification and depletion under chronic activation.

Factor D, also known as adipsin, initiates alternative-pathway amplification by activating Factor B to assemble the C3bBb convertase. Unlike the declining trajectories observed for C4, C4b, Factor I and Properdin, Factor D levels were consistently elevated in AD. The sustained elevation of Factor D may indicate that its role in AD extends beyond alternative-pathway amplification, potentially also reflecting adipose-derived metabolic and inflammatory pathways. Factor D is secreted predominantly by adipocytes and has been implicated in systemic energy balance, pancreatic β-cell function and insulin secretion [39]. Elevated plasma Factor D has been associated with obesity, dyslipidemia, and type 2 diabetes [40, 41], as well as vascular dysfunction and systemic inflammation [42]. In the CNS, Factor D increases with advancing age and correlates with inflammatory markers [43]. Persistent elevation of Factor D in AD may therefore reflect a convergence of complement activation, metabolic dysregulation and chronic inflammation, processes that are increasingly recognized as central to AD pathogenesis.

Taken together, these data indicate that complement dysregulation in preclinical AD simultaneously engages the classical/lectin axis (C4, C4b, and Factor I) and the alternative pathway (Properdin and Factor D). Notably, this longitudinal cohort predominantly represents late-onset AD, with a slowly progressive course. In this context, relatively modest plasma complement shifts may be compatible with chronic low-grade consumption and protein “burnout”, rather than overt systemic hyperactivation. The distinct longitudinal patterns, with declines in C4, C4b, Factor I and Properdin alongside a sustained increase in Factor D, argue against a unitary pathway explanation and instead suggest coordinated perturbation across multiple complement axes. Consistent with this pathway-spanning pattern, ROC analyses showed that a five-complement-factor panel integrating markers from the classical/lectin axis and the alternative pathway achieved better discrimination than any single factor, with additional improvement after inclusion of *APOE ε4* status, supporting the value of pathway-informed multi-marker integration for identifying preclinical AD.

This study has several limitations. First, our analysis was based primarily on complement measurements in peripheral plasma. Although peripheral biomarkers are advantageous for their accessibility and translational potential, they may not accurately reflect complement activity within the CNS. Circulating complement profiles are likely to approximate central processes only when blood-brain barrier (BBB) integrity is compromised, a phenomenon increasingly recognized in AD. Thus, future studies combining CSF biomarkers, advanced neuroimaging, and measures of BBB permeability will be important to clarify the extent to which peripheral complement alterations track CNS complement activity. Second, although the longitudinal design allowed us to capture temporal patterns of complement change, causality cannot be inferred from these observational data. Complement dysregulation and core AD pathologies, such as Aβ and tau, may influence each other bidirectionally. Clarifying these mechanistic interactions will be important for the development of targeted therapeutic strategies. Third, lifestyle-related factors such as diet, physical activity, smoking and alcohol consumption, which are known to modulate systemic immune function, were not systematically incorporated into the present analyses. These behaviors may influence circulating complement levels, and residual confounding by lifestyle factors therefore cannot be fully excluded. Future work integrating detailed lifestyle information with longitudinal complement profiling will be important to disentangle the contributions of behavioral risk factors from disease-related immune alterations. Fourth, the sample size was relatively modest, in part because the COVID-19 pandemic affected long-term follow-up, potentially reducing power to detect more subtle effects. Finally, studies with longer follow-up periods and broader methodological approaches are needed to validate these observations and elucidate the underlying mechanisms linking complement dysregulation to the progression of AD.

In conclusion, we show that longitudinal dysregulation of complement factors, including C4, C4b, Factor I, Factor D and Properdin, emerges before the onset of AD symptoms and is closely associated with disease progression. These findings highlight the complement system as a promising early therapeutic target in AD and support the development of precision medicine strategies that incorporate complement-based signatures to optimize timing and selection of early interventions. Further studies with extended follow-up and broader methodological approaches remain warranted to validate these observations and elucidate the mechanisms linking complement dysregulation to AD progression.

## Methods

### Study design and participants

In this study, we included two cohorts: a longitudinal cohort (cohort 1) of participants who were cognitively normal at baseline 10 years before the study and a cross-sectional cohort (cohort 2) comprising participants with various types of dementia. We continuously followed participants in cohort 1 (controls, *n* = 118; Pre-AD, *n* = 117) and conducted comprehensive clinical assessments and blood sample collection every 2 years to evaluate changes in complement levels over 10 years. To identify AD-specific complement factors, we included patients with different types of dementia in cohort 2 (control, *n* = 57; AD, *n* = 53; VaD, *n* = 54; PDD, *n* = 51; bvFTD, *n* = 56; and DLB, *n* = 52). AD was clinically diagnosed following the 2011 guidelines established by the National Institute on Aging and Alzheimer’s Association [44]. To further distinguish AD from controls, we applied the biomarker criteria, CSF phosphorylated tau (P-tau181)/Aβ42 > 0.14 and CSF Aβ42 < 500 pg/mL, based on our previously published findings, which were corroborated by other studies [45]. The diagnoses of VaD, PDD, bvFTD, and dementia with DLB followed the established criteria [46–49]. Cognitively healthy participants matched by age and sex served as controls. All participants underwent standardized clinical screening, and individuals with acute infections, clinically diagnosed autoimmune or other chronic inflammatory diseases, active malignancies, or recent major surgeries or traumas were excluded or temporarily deferred. This study was approved by the Institutional Review Board of Xuanwu Hospital, Capital Medical University. Prior to enrollment, all participants and their legal guardians provided informed consent.

### Cerebrospinal fluid collection and analysis

CSF samples were collected via lumbar puncture and processed according to a standardized procedure [50]. The participant was placed in the left lateral decubitus position, and a 20-gauge atraumatic needle was used to obtain 15 mL of CSF from the intervertebral spaces between L3 and L5. Subsequently, the CSF samples were centrifuged at 2000 × g for 10 min at room temperature, aliquoted, and stored in polypropylene tubes at -80 °C. The levels of Aβ42, total tau (T-tau), and P-tau181 in the CSF were measured using commercially available ELISA kits (Fujirebio, Tokyo, Japan).

### Blood collection and protein measurement

Participants fasted for 12 h before blood collection. Blood was drawn into EDTA-containing tubes and processed immediately by centrifugation at 4200 × g for 10 min. Plasma was aliquoted without delay and stored at -80 °C according to standardized protocols. To minimize degradation, each sample underwent only one freeze-thaw cycle before analysis. To further reduce pre-analytical variability, all samples were assayed simultaneously at the 10th year of follow-up using a single lot of reagents, thereby minimize batch-to-batch variation.

Plasma complement levels were quantified using multiplex bead-based xMAP technology (Luminex Corp., Northbrook, IL) and commercially available kits (EMD Millipore, Milliplex Map, Burlington, MA). Fourteen complement factors spanning the three activation pathways and the terminal pathway were analyzed. The MILLIPLEX MAP Human Complement Panel 1 was used to measure C2, C4b, C5, C5a, C9, MBL, Factor D, Factor I, and Properdin; Panel 2 was used to measure C1q, C3, C4, Factor B, and Factor H; and C5b-9 was measured using the MILLIPLEX MAP Human Complement C5b-9 kit. Assays were performed on a Luminex 200 instrument according to the manufacturer’s instructions. hsCRP was determined using an automatic biochemical analyzer (Hitachi 7600, Hitachi Ltd., Tokyo, Japan). All samples were assayed in duplicate, and mean values were reported. Raw multiplex outputs were expressed as mean fluorescence intensity, and absolute concentrations were derived from standard curves using a four-parameter logistic regression model. Three quality controls (QCs), including at least one laboratory-developed and validated QC, were included in each run.

### Statistical analysis

*χ²* tests were used to analyze categorical variables such as sex and *APOE ε4* status. Student’s t tests were applied to continuous variables (e.g., age, years of education, MMSE scores, core AD biomarkers and complement factor levels), with analysis of variance used when more than two groups were compared. Linear mixed-effects regression models (lme4 package in R) were used to assess longitudinal changes in complement levels. Linear regression models were used to evaluate the cross-sectional correlations between complement factors and core AD biomarkers. Model accuracy was assessed through receiver operating characteristic (ROC) curve analysis, and multicollinearity among predictors was examined by calculating tolerance and variance inflation factor (VIF) statistics. The statistical significance threshold was set at *P* < 0.05. All analyses were conducted using SPSS Statistics for Windows, version 22 (IBM) and R software version 4.2.3 (R Foundation for Statistical Computing).

## Supplementary Information

Below is the link to the electronic supplementary material.

Supplementary Material 1

## Data Availability

The data supporting the findings of this study can be obtained by contacting the corresponding author upon request.
