# Supplementary material for: Systemic complement factors in aging, Alzheimer’s disease and other dementias: a longitudinal study over 10 years
Source: Mol Neurodegener. 2026 Jan 12;21:11. doi: 10.1186/s13024-026-00927-3 (PMC12888596; doi:10.1186/s13024-026-00927-3)
Supplement: Supplementary file 1 — Supplementary Material 1 [file 13024_2026_927_MOESM1_ESM.pdf]

Supplementary Material

Title:

Systemic complement factors in aging, Alzheimer's disease and other dementias: a longitudinal study over 10 years

The Supplementary materials include the following information:

1. **Supplementary Fig. 1: Prediction of complement C4, C4b, Factor D, Factor I, and Properdin for AD at the 10-year follow-up.**
2. **Supplementary Fig. 2: Prediction of complement C4, C4b, Factor D, Factor I, and Properdin for preclinical AD at baseline.**
3. **Supplementary Fig. 3: Plasma complement factor levels stratified by *APOE ε4* status in controls and individuals with preclinical AD.**
4. **Supplementary Fig. 4: Plasma complement factor levels stratified by sex in controls and individuals with preclinical AD.**
5. **Supplementary Figs. 5-18: Levels of complement factors in participants with AD and controls at baseline and follow-ups.**
6. **Supplementary Fig. 19: Longitudinal trajectories of plasma complement factors without significant group differences.**
7. **Supplementary Fig. 20: Complement factors without significant differences in various types of dementia.**
8. **Table S1. Values of other markers.**

1. Supplementary Fig. 1

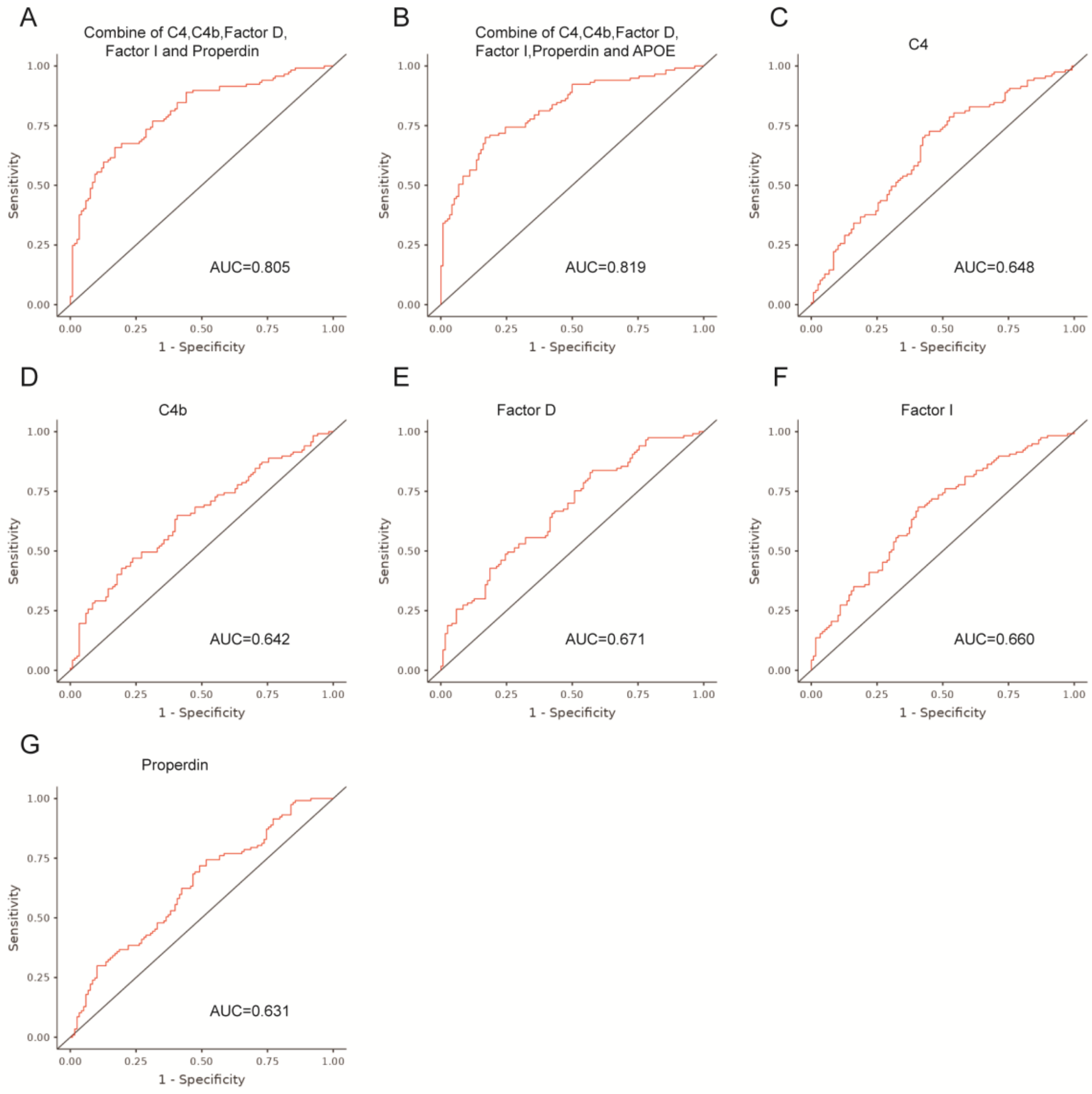

Supplementary Fig. 1 Prediction of complement C4, C4b, Factor D, Factor I, and Properdin for AD at the 10-year follow-up. ROC curve analysis and corresponding AUCs from logistic regression models for complement C4, C4b, Factor D, Factor I, and Properdin to assess accuracy when differentiating participants with AD from cognitively normal controls at 10-year follow-up. Models were generated from a combination of complement C4, C4b, Factor D, Factor I, and Properdin with (A) or without (B) *APOE ε4* status and the individual markers (C-G). n = 118 (controls), 117 (AD). APOE, apolipoprotein E; AUC, areas under the curve; AD, Alzheimer's disease.

2. Supplementary Fig. 2

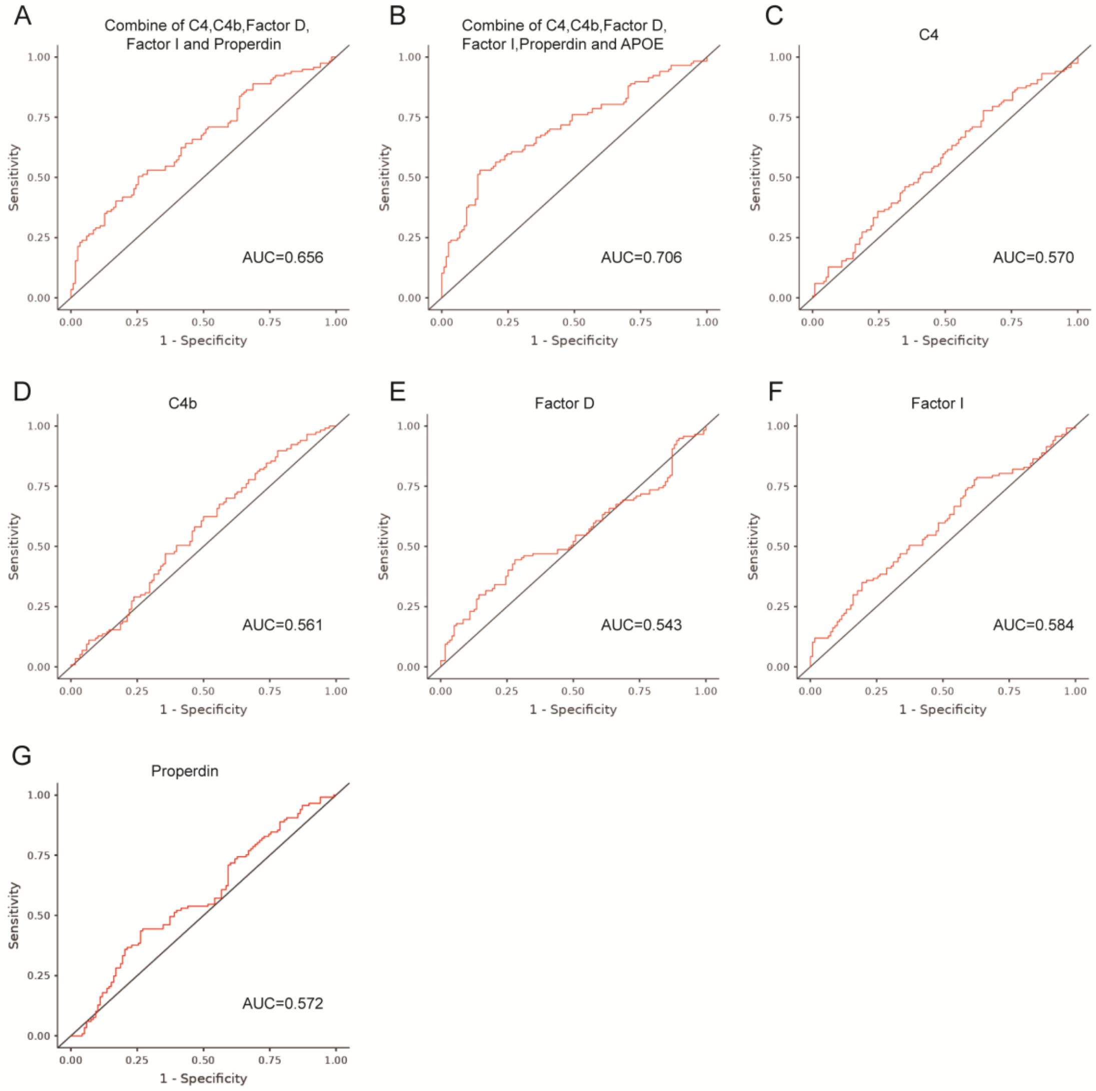

Supplementary Fig. 2 Prediction of complement C4, C4b, Factor D, Factor I, and Properdin for preclinical AD at baseline. ROC curve analysis and corresponding AUCs from logistic regression models for complement C4, C4b, Factor D, Factor I, and Properdin to assess accuracy when differentiating participants with preclinical AD from cognitively normal controls at baseline. Models were generated from a combination of complement C4, C4b, Factor D, Factor I, and Properdin with (A) or without (B) *APOE ε4* status and the individual markers (C-G). n = 118 (controls), 117 (preclinical AD). APOE, apolipoprotein E; AUC, areas under the curve; AD, Alzheimer's disease.

3. Supplementary Fig. 3

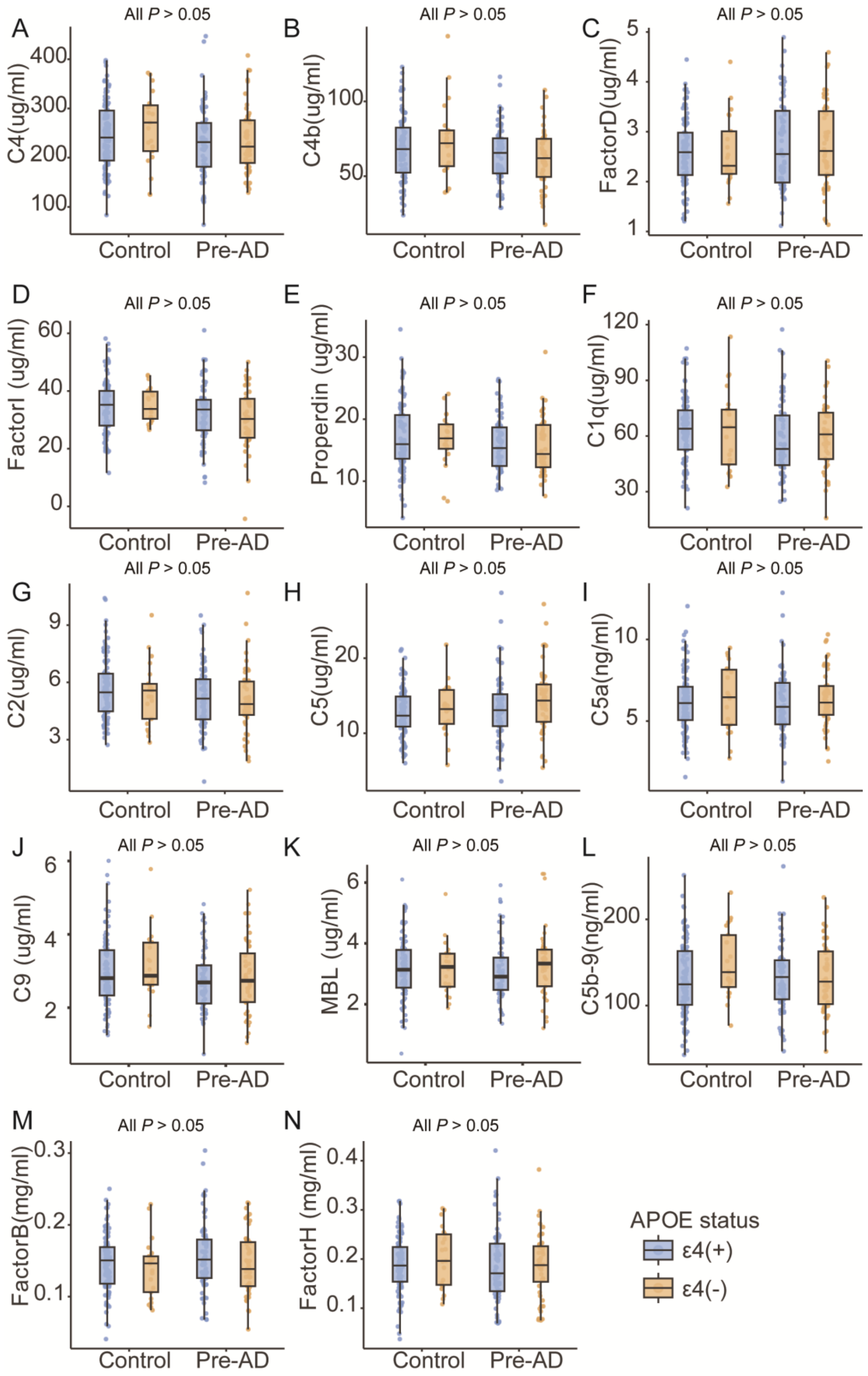

Supplementary Fig. 3 Plasma complement factor levels stratified by *APOE ε4* status in controls and individuals with preclinical AD. Plasma concentrations of (A) C4, (B) C4b, (C) Factor D, (D) Factor I, (E) Properdin, (F) C1q, (G) C2, (H) C5, (I) C5a, (J) C9, (K) MBL, (L) C5b-9, (M) Factor B and (N) Factor H in cognitively normal controls and individuals with Pre-AD, further stratified by *APOE ε4* carrier status [ε4(+) (blue) and ε4(–) (yellow)]. No significant differences in any complement factor were observed between APOE ε4 carriers and non-carriers within either group (all $P > 0.05$). n = 118 (controls), 117 (Pre-AD). Pre-AD, preclinical Alzheimer's disease; APOE, apolipoprotein E; MBL, mannose-binding lectin.

4. Supplementary Fig. 4

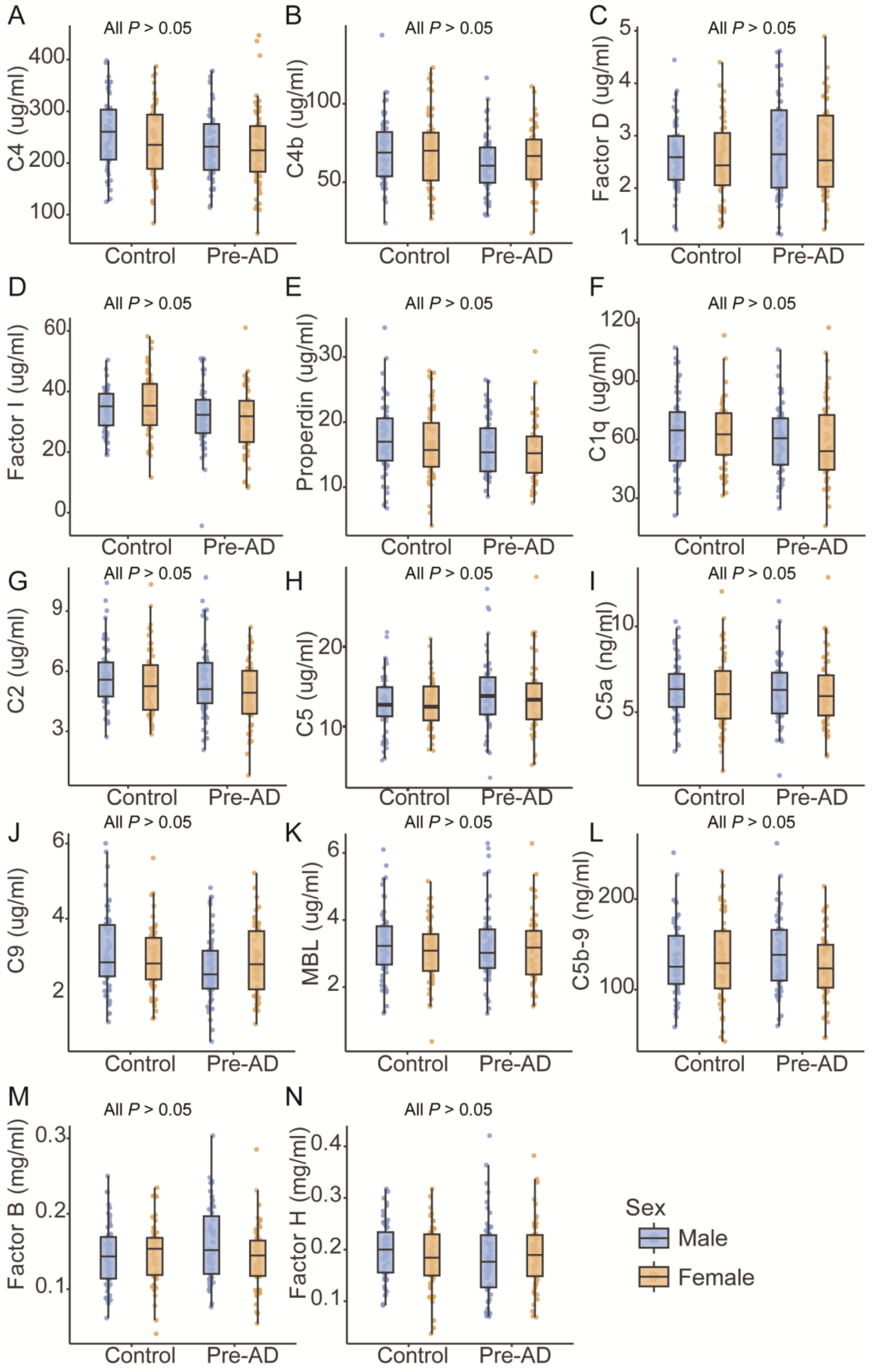

Supplementary Fig. 4 Plasma complement factor levels stratified by sex in controls and individuals with preclinical AD. Plasma concentrations of (A) C4, (B) C4b, (C) Factor D, (D) Factor I, (E) Properdin, (F) C1q, (G) C2, (H) C5, (I) C5a, (J) C9, (K) MBL, (L) C5b-9, (M) Factor B and (N) Factor H in cognitively normal controls and individuals with Pre-AD, stratified by sex [male (blue) and female (yellow)]. No significant sex-related differences in any complement factor were observed within either diagnostic group (all P > 0.05). n = 118 (controls), 117 (Pre-AD). Pre-AD, preclinical Alzheimer's disease; MBL, mannose-binding lectin.

5. Supplementary Fig. 5-18

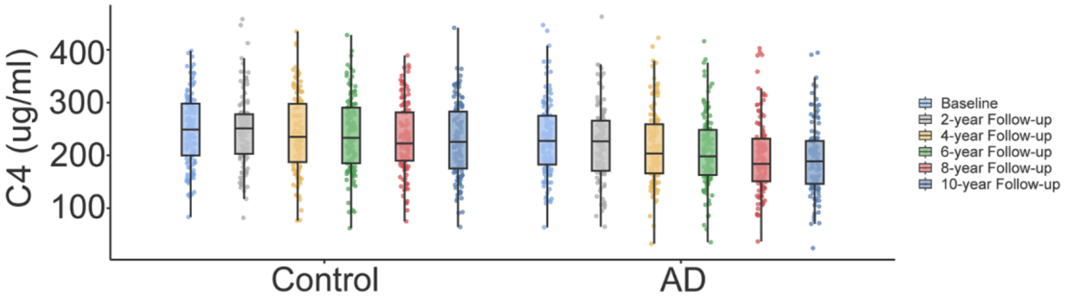

Supplementary Fig. 5 Levels of complement C4 in participants with AD and controls at baseline and follow-ups. n = 118 (controls), 117 (AD). AD, Alzheimer's disease.

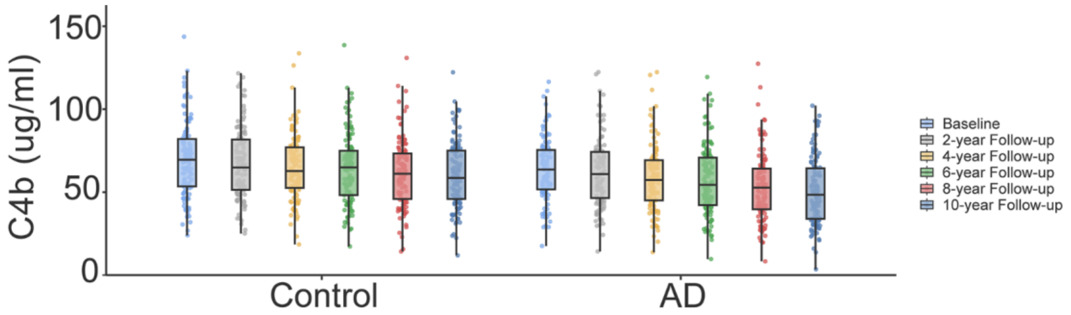

Supplementary Fig. 6 Levels of complement C4b in participants with AD and controls at baseline and follow-ups. n = 118 (controls), 117 (AD). AD, Alzheimer's

disease.

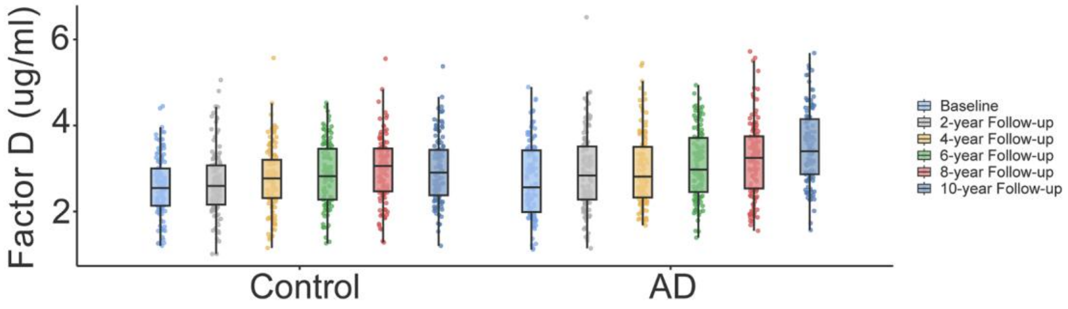

Supplementary Fig. 7 Levels of complement Factor D in participants with AD and controls at baseline and follow-ups. n = 118 (controls), 117 (AD). AD, Alzheimer's disease.

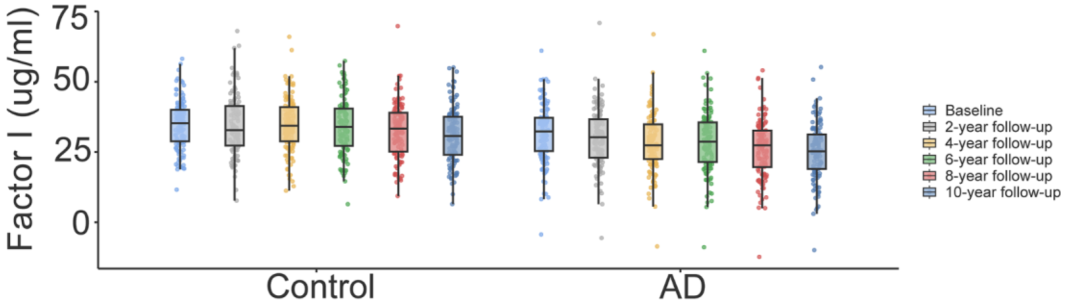

Supplementary Fig. 8 Levels of complement Factor I in participants with AD and controls at baseline and follow-ups. n = 118 (controls), 117 (AD). AD, Alzheimer's disease.

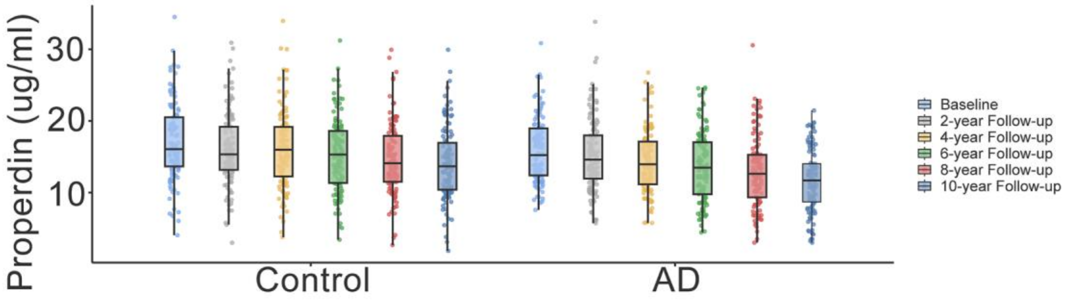

Supplementary Fig. 9 Levels of Properdin in participants with AD and controls at baseline and follow-ups. n = 118 (controls), 117 (AD). AD, Alzheimer's disease.

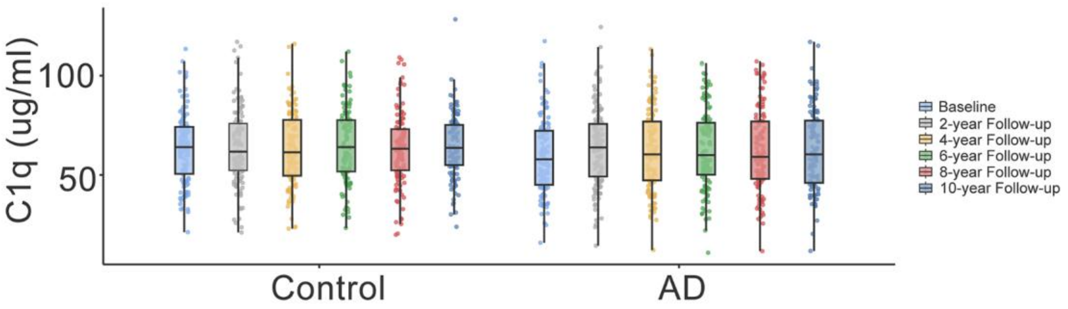

Supplementary Fig. 10 Levels of complement C1q in participants with AD and controls at baseline and follow-ups. n = 118 (controls), 117 (AD). AD, Alzheimer's disease.

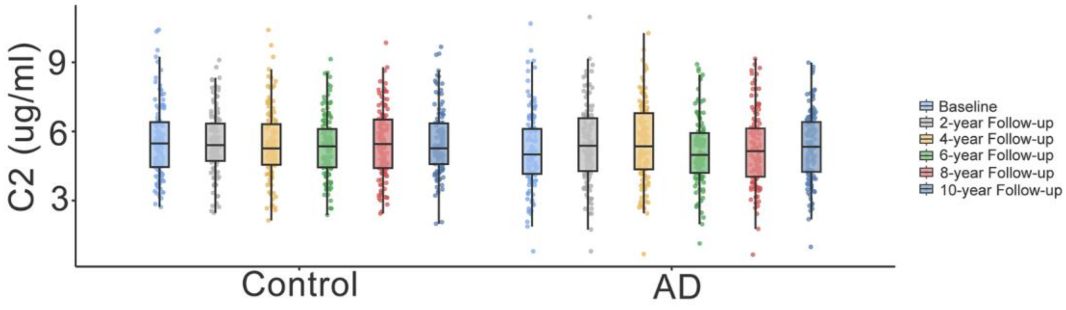

Supplementary Fig. 11 Levels of complement C2 in participants with AD and controls at baseline and follow-ups. n = 118 (controls), 117 (AD). AD, Alzheimer's disease.

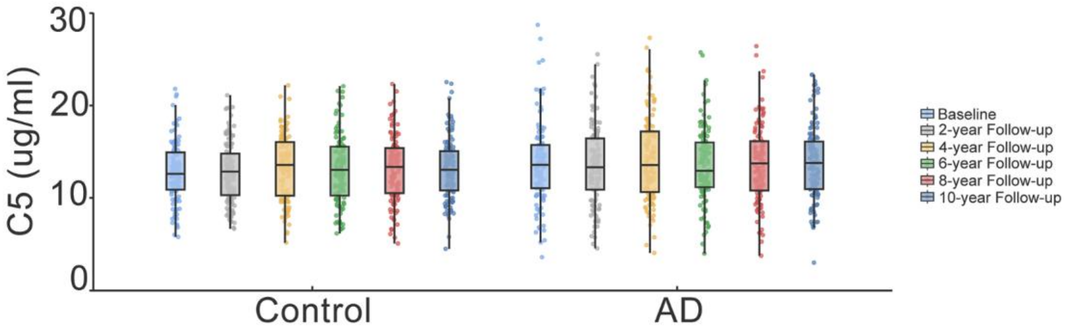

Supplementary Fig. 12 Levels of complement C5 in participants with AD and

controls at baseline and follow-ups. n = 118 (controls), 117 (AD). AD, Alzheimer's disease.

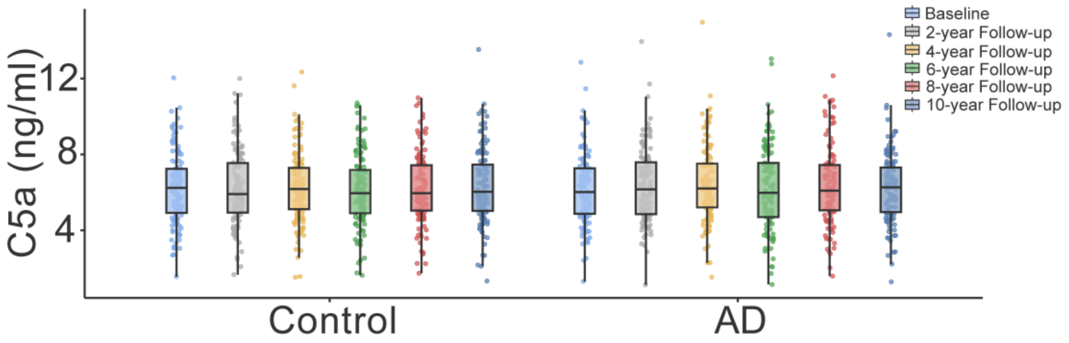

Supplementary Fig. 13 Levels of complement C5a in participants with AD and controls at baseline and follow-ups. n = 118 (controls), 117 (AD). AD, Alzheimer's disease.

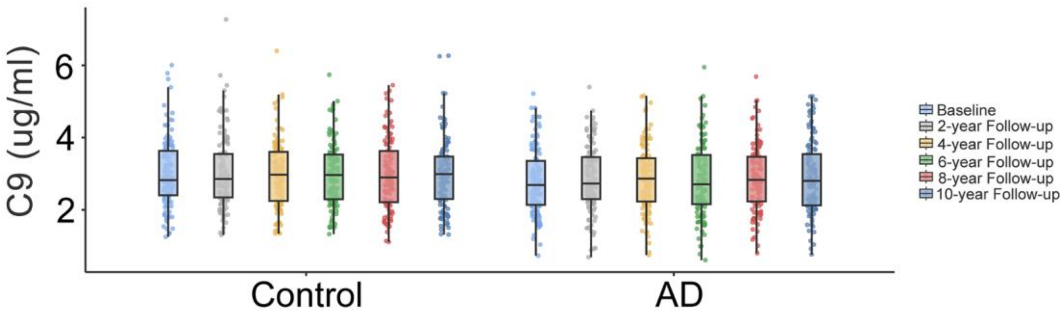

Supplementary Fig. 14 Levels of complement C9 in participants with AD and controls at baseline and follow-ups. n = 118 (controls), 117 (AD). AD, Alzheimer's disease.

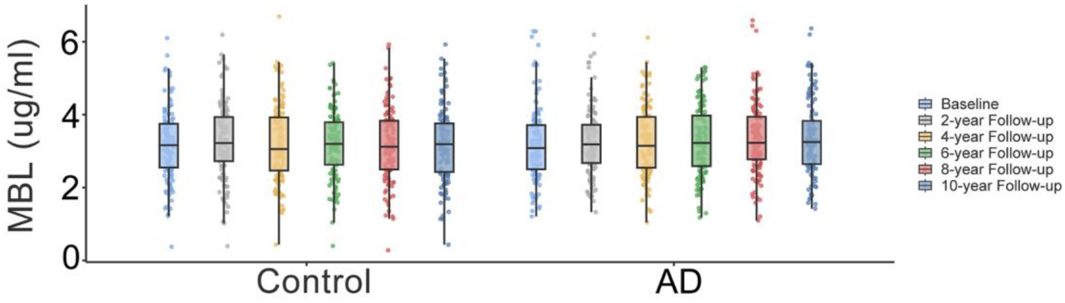

Supplementary Fig. 15 Levels of MBL in participants with AD and controls at baseline and follow-ups. n = 118 (controls), 117 (AD). AD, Alzheimer's disease; MBL, mannose-binding lectin.

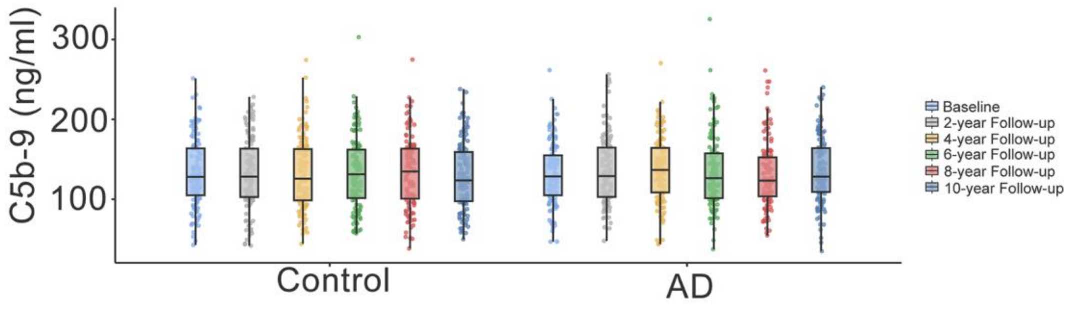

Supplementary Fig. 16 Levels of C5b-9 in participants with AD and controls at baseline and follow-ups. n = 118 (controls), 117 (AD). AD, Alzheimer's disease; C5b-9, complement membrane attack complex.

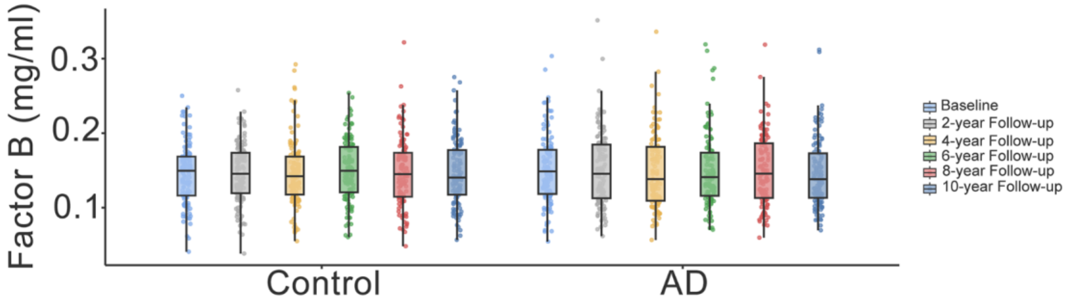

Supplementary Fig. 17 Levels of complement Factor B in participants with AD and controls at baseline and follow-ups. n = 118 (controls), 117 (AD). AD, Alzheimer's disease.

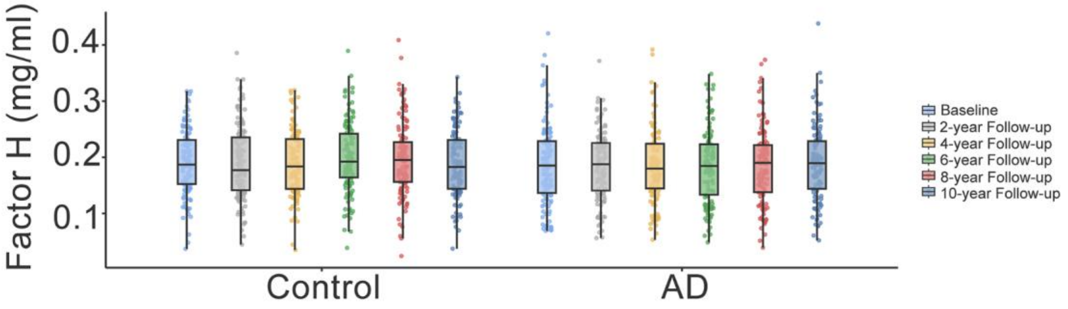

Supplementary Fig. 18 Levels of complement Factor H in participants with AD and controls at baseline and follow-ups. n = 118 (controls), 117 (AD). AD, Alzheimer's disease.

2. Supplementary Fig. 19

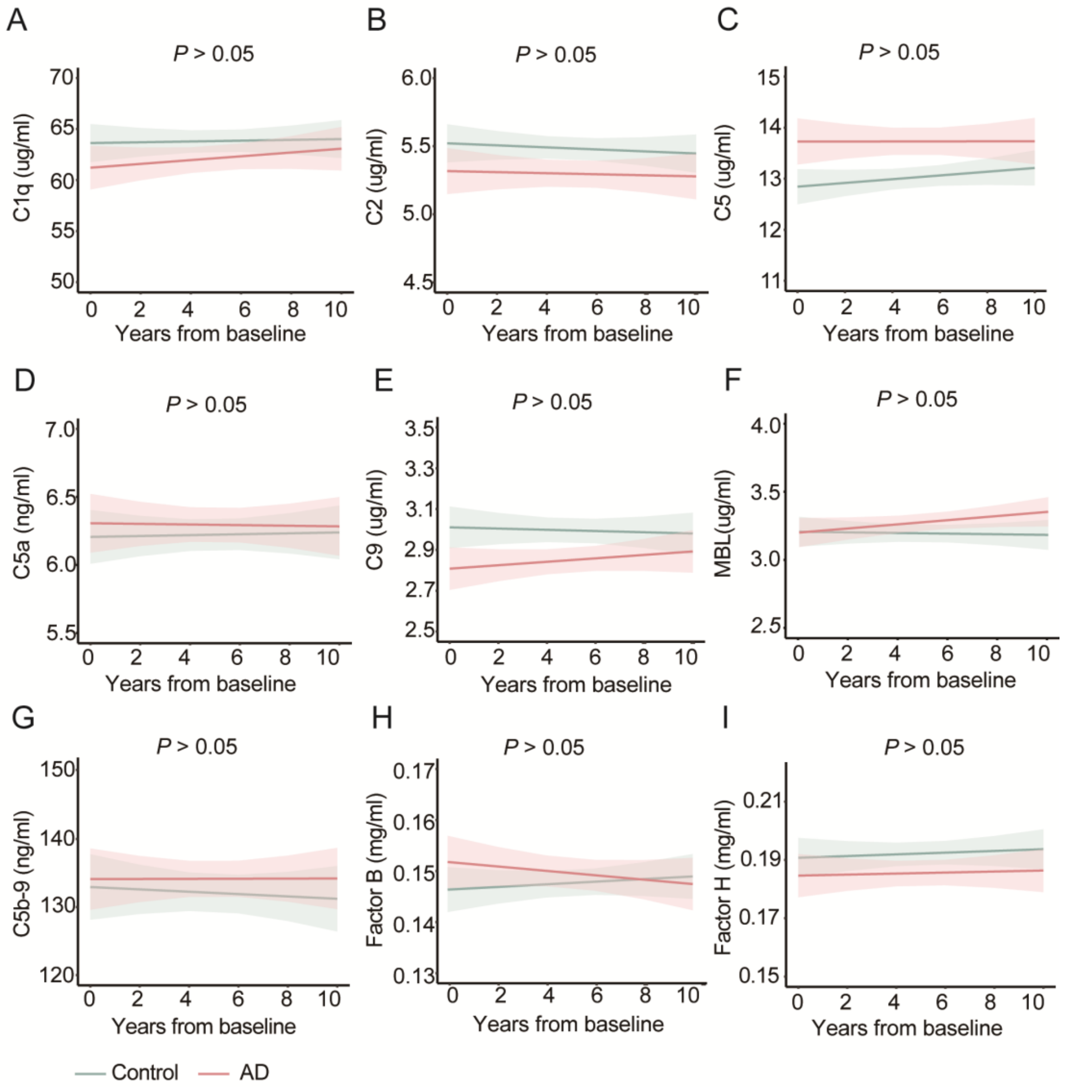

Supplementary Fig. 19 Longitudinal trajectories of plasma complement factors without significant group differences. Longitudinal trajectories of plasma complement C1q (**A**), C2 (**B**), C5 (**C**), C5a (**D**), C9 (**E**), MBL (**F**), C5b-9 (**G**), Factor B (**H**), and Factor H (**I**) are shown by diagnostic group. No statistically significant differences were observed between participants with AD and cognitively normal controls over time. The x-axis indicates time since baseline. Shaded areas represent 95% confidence intervals of regression lines derived from linear mixed-effects models

evaluating the interaction between diagnostic status and follow-up duration. AD, Alzheimer's disease; C5b-9, complement membrane attack complex; MBL, mannose-binding lectin.

3. Supplementary Fig. 20

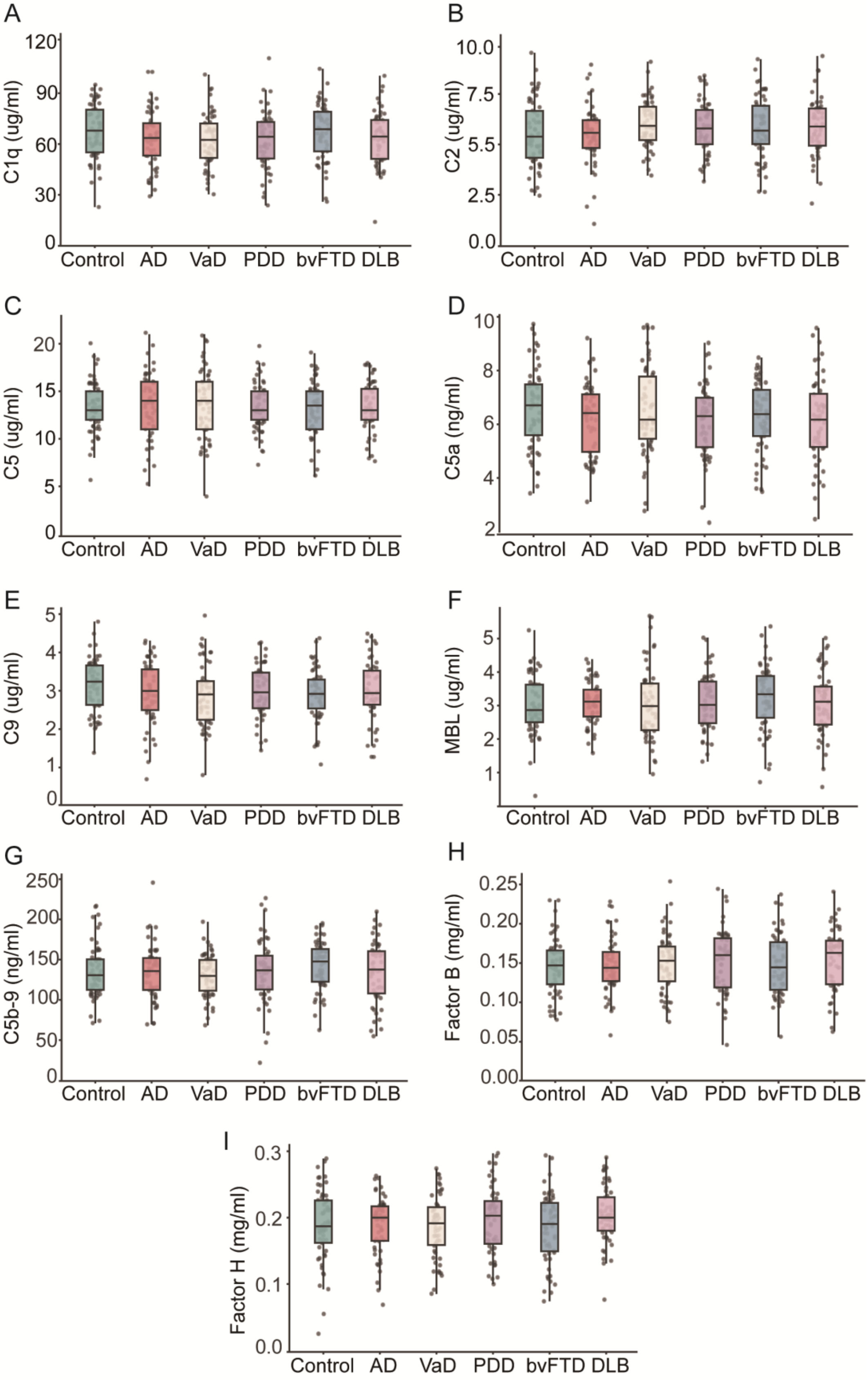

Supplementary Fig. 20 Complement factors without significant differences in various types of dementia. The levels of complement C1q (**A**), C2 (**B**), C5 (**C**), C5a (**D**), C9 (**E**), MBL (**F**), C5b-9 (**G**), Factor B (**H**), and Factor H (**I**) in cohort 2. No statistically significant differences were observed between participants with various dementia subtypes and cognitively normal controls. The levels of the complement factors were compared between participants with different types of dementia and cognitively normal controls using the *t*-test. $n = 57$ (controls), 53 (AD), 54 (VaD), 51 (PDD), 56 (bvFTD), 52 (DLB). AD, Alzheimer's disease; bvFTD, behavioural variant frontotemporal dementia; C5b-9, complement membrane attack complex; DLB, dementia with Lewy bodies; MBL, mannose-binding lectin; PDD, Parkinson's disease dementia; VaD, vascular dementia.

Table S1. Values of other markers.

Markers	Baseline			At the 10-year follow-up		
	Control (n = 118)	Pre-AD (n =117)	*P* value	Control (n = 118)	AD (n = 117)	*P* value
C3 (mg/mL)	1.5 (0.26)	1.2 (0.20)	< 0.05	1.3 (0.25)	0.9 (0.17)	< 0.05
hsCRP (mg/L)	1.6 (0.29)	1.7 (0.30)	> 0.05	1.5 (0.28)	1.6 (0.27)	> 0.05

The values of markers are shown as mean (SD). Differences between the groups were tested using Student's t-test. Abbreviations: AD, Alzheimer's disease; Pre-AD, preclinical Alzheimer's disease; SD, standard deviation. hsCRP was measured using a Hitachi 7600 Automatic Biochemical Analyzer.
